# Effect of Tea Theaflavins and Catechins on Microvascular Function

**DOI:** 10.3390/nu6125772

**Published:** 2014-12-11

**Authors:** Dagmar Fuchs, Young de Graaf, Roeland van Kerckhoven, Richard Draijer

**Affiliations:** 1 Unilever Research & Development, 3133 AT Vlaardingen, The Netherlands; E-Mails: young-de.graaf@unilever.com (Y.G.); richard.draijer@unilever.com (R.D.); 2 RVK Research & Consulting BVBA, 2180 Ekeren, Belgium; E-Mail: Roelandvankerckhoven@yahoo.com

**Keywords:** tea, theaflavins, catechins, polyphenols, vascular function, EndoPAT, reactive hyperemia response

## Abstract

Beneficial effects of flavonoid-rich black and green tea on macrocirculation have been well established. Theaflavins are unique to black tea as they are formed from catechins during the enzymatic oxidation of tea leaves. The study was performed to gain more insight into the effects of theaflavins on microcirculation and to compare effects with another important flavonoid class, the green tea derived catechins, which have been reported to improve vascular function. Twenty-four healthy subjects were included in a double-blind, placebo-controlled, randomised, cross-over study. On six different days, subjects received capsules with a single dose of catechins (500 mg), four varying doses of theaflavins (100 to 500 mg) or placebo. Microcirculation was assessed after each treatment by Pulse Amplitude Tonometry (EndoPAT) at baseline and 2, 4 and 6 h after test product intake. The EndoPAT reactive hyperemia response was improved by 500 mg catechins (reactive hyperemia index (RHI): 0.2; *p* = 0.04) and by 500 mg theaflavins (RHI: 0.19; *p* = 0.06) compared to placebo. Also, 300 mg theaflavins increased the RHI (0.28; *p* = 0.02), but no effects were observed at lower doses. The study suggests moderate effects of single doses of catechins and theaflavins on peripheral microcirculation.

## 1. Introduction

Tea made of the leaves from *Camellia sinensis* is considered to be the second most frequently consumed beverage worldwide after water [1]. About 50% of the total dietary intake of flavonoids are derived from tea, which makes it to the most important dietary source thereof in the western world [2].

An inverse association between the consumption of tea and cardiovascular events has been observed in several epidemiological studies and the evidence is growing that these beneficial effects of tea may be mediated by its flavonoid components [3]. The reported strength of the associations between black and green tea and health outcomes does to some extent differ between analyses [4]. One important shortcoming is that the tea composition may differ considerably due to tea plant variaties, conditions of growth and brewing procedure. Moreover, the exact tea (flavonoid) composition is usually not reported or unknown. Identification of the actives of tea is therefore warranted. There are implications that tea flavonoids exert their beneficial effects via several mechanisms such as anti-proliferative, anti-inflammatory, and anti-thrombogenic properties, but also via favourable effects on endothelial function and blood pressure. Particularly, the effect of black and green tea on endothelial function, as assessed by flow-mediated vasodilation (FMD), is convincing [5]. The evidence is growing that catechins, such as epicatechin and epigallocatechin-gallate, are responsible for the effects of tea on cardiovascular health [6]. Although the catechin concentration in green tea is much higher compared to black tea [7], green tea and black tea show similar sized effects on FMD [8]. This suggests that other (flavonoid) actives or metabolites thereof may be responsible for the observed effects of black tea. Theaflavins are among the most obvious candidates, being unique to black tea, and representing about 10% of the black tea flavonoids [7]. Theaflavins are formed in black tea leaves from catechins during the “fermentation” named enzymatic oxidation [7]. Based on the gallate substitution and position theaflavins can be subdivided into theaflavin, theaflavin-3-monogallate, theaflavin-3′-monogallate and theaflavin-3,3′-digallate [6].

While the efficacy to improve endothelial function is well established for black tea [5] the exact contribution of the black tea theaflavins remains to be elucidated. Studies with ApoE gene-knockout mice revealed that theaflavin consumption may attenuate atherosclerosis by improving NO bioavailability [9], but human data are lacking. The evidence for black tea benefits on vascular health in humans is mostly based on FMD measurements in large arteries [5]. A meta-analysis including all data available for the effects of tea on vascular function as measured by FMD revealed that the consumption of about 500 mL of tea, which corresponds to 2–3 cups of tea per day, improves FMD by on average 2.6% [5]. The FMD value, which is associated with future cardiovascular events [10], depends on the availability of nitric oxide [11]. Vascular function may also be assessed by Pulse Amplitude Tonometry (EndoPAT), which partly depends on the endothelial nitric oxide release [11] and has been associated with cardiovascular risk factors [12]. The EndoPAT measurement determines changes in the pulse amplitude in the finger which reflects changes in digital flow and digital vessel dilation during reactive hyperemia. It has been shown that the results of EndoPAT measurements correlate modestly, but significant with brachial artery FMD measurements [13,14]. Ingestion of green tea has been shown to affect reactive hyperemia responses as measured by EndoPAT [15], but in particular beneficial effects of cocoa-derived flavonoids have been reported on vascular function determined by EndoPAT [16,17,18,19] and FMD measurements [19]. As the FMD and EndoPAT measurement determine effects that are at least in part mediated by the release of nitric oxide [11] we hypothesized that the established black and green tea effects on FMD [5] should be reflected in EndoPAT readings as marker for microvascular function.

Therefore the aim of the current study was to gain more insight on the acute effects of theaflavins on micrcocirculation and to compare these effects with those of a green tea derived catechin treatment. Additionally, formation and identification of metabolites of catechins and theaflavins was studied using different analytical methods.

## 2. Experimental Section

### 2.1. Study Population

The present study was conducted according to the guidelines laid down in the Declaration of Helsinki and all procedures involving human subjects were approved by the Medical Ethics Committee of Wageningen University, The Netherlands. The study was conducted according to the Dutch Medical Research Involving Human Subjects Act and the International Conference on Harmonization Guidelines for Good Clinical Practice. The study took place from November to December 2008 at Unilever Research & Development Vlaardingen, The Netherlands. Written informed consent was obtained from all subjects.

The subject sample size was calculated with an alpha of 0.05 (two-sided) and a power of 80% and a within subject variation of the reactive hyperemia index (RHI) response of 0.128 (based on internal unpublished data) indicated that 20 subjects should be sufficient to detect a difference of 0.34 in the RHI between two different dosed treatments (placebo *vs.* maximum dose). In order to account for drop outs, 24 subjects were recruited into this study. An independent statistician randomized the subject codes to one of the six treatment sequences according to a Williams design. During the study, the key revealing the p-codes related to the treatments was only known by the two persons involved in preparing the test and placebo products. For cases of emergency, two sets of sealed envelopes with the subjects’ code-treatment combination were kept at the test facility. These envelopes were not opened during the study. After a blind review of the results at the end of the study and analysis of the data, the code was broken.

Apparently healthy men and postmenopausal women aged 45–75 years with a body mass index of 19 to 30 kg/m^2^ were recruited among the inhabitants of Vlaardingen by pre-selection from a database. Women had to be postmenopausal (*i.e.*, not menstruating) for at least one year and not taking hormone replacement therapy within one year before the start of the intervention. A total of 268 persons were interested in joining one of the information sessions. Because of the large amount of interested persons, only 175 persons were invited for the information meeting and the other 93 subjects were excluded by lot. Of these 175 persons, 150 were present at one of the two information sessions and 147 persons filled in a selection questionnaire which covered a number of inclusion and exclusion criteria. Based on this questionnaire 89 persons were invited for a screening visit. In total 58 persons were excluded. The main exclusion criteria were habitual tea consumption and use of medication that could interfere with the outcome parameters. Prior to the start of the screening 2 subjects withdrew from further participation. A total of 33 subjects were excluded based on the results of the screening (*i.e.*, high blood pressure, deviations in dipstick urine analysis of protein and glucose, *etc.*). Of the 54 eligible subjects, 27 were excluded by lot. Twenty-four subjects and three reserve subjects—to replace possible drop-outs—started the run in period.

The general health of the subjects was determined by means of the selection questionnaire at the information meeting, and by taking urine and blood samples on the morning of the screening appointment. Subjects came to the test facility and handed in some morning urine for dipstick analysis of protein and glucose in urine. Body weight, height and body mass index (BMI) (calculated as weight in kilograms divided by height in meters squared) were determined and their office blood pressure was measured. For analysis of the routine parameters two blood samples (a 2 mL Ethylene Diamine Tetraacetic Acid (EDTA) tube for a complete blood cell count and a 5 mL gel tube for liver function, renal function, and inflammatory function) were collected for clinical chemistry.

The study was not registered in one of the clinical trials registers.

### 2.2. Study Design

The study was conducted with a double-blind, placebo controlled, randomised, cross-over design in which the acute effect of varying doses of theaflavins and a single dose of catechins compared to placebo on microcirculation was measured. Between the different treatments a wash-out period of 6 days was provided. On each visit day, subjects ingested the specific treatment at the test facility subsequent to the baseline measurement (fasting state). Subjects were asked to reduce their consumption of flavonoid-rich foods and beverages and to refrain from using aspirin, tea, alcohol and coffee on the day prior to the measurement day because of interference with the outcome measurements. At the visit days, PAT readings were performed in fasted state and 2, 4 and 6 h after test product intake. One and five hours after the ingestion of the test products the subjects had a standardized low-calorie meal. To make it easier for the subjects to comply with the dietary restrictions, snacks and a standardized diner were provided for the day prior to the test day. On the test day breakfast, lunch, drinks, snacks and diner were provided as well. In addition, subjects registered compliance to background diet for the days with dietary restrictions (*i.e.*, the visit days and days prior to a visit) on a special form.

### 2.3. Test Products

Five active test products and one control product in the form of capsules were given to the subjects. Gelatine capsules size 0 (Capsugel, Bornem, Belgium) were filled with green tea extract equivalent to 100 mg of catechins (Sunphenon 90DCF-T, Taiyo Kagaku, Tokyo, Japan), theaflavin-rich black tea extract equivalent to 100 mg theaflavins (Unilever R & D Colworth, Sharnbrook, Bedfordshire, England) or microcrystalline cellulose (FMC BioPolymer, Wallingstown, Cork, Ireland) as placebo. The theaflavins and catechins capsules contained microcrystalline cellulose as additional filling material. For each treatment the subjects received 5 capsules. For the varying doses of theaflavins subjects received the equivalent number of capsules filled with theaflavin and the remaining capsules were filled with microcrystalline cellulose. For example the treatment with 200 mg theaflavins consisted of 2 capsules filled with theaflavins and 3 capsules filled with microcrystalline cellulose. The green tea extract Sunphenon 90DCF-T represented a mixture of catechins with a total polyphenol content of 95.8%, a total catechin content of 83.2% and an epigallocatechingallate content of 54.4% (data derived from suppliers’ certificate of analysis). The theaflavin-rich black tea extract contained 81% total theaflavins which consisted of 10% theaflavin, 25% theaflavin monogallate, 19% theaflavin-3′-monogallate and 27% theaflavin digallate (data derived from reverse phase high-performance liquid chromatography (HPLC) separation and nuclear magnetic resonance (NMR) quantification done by Unilever).

### 2.4. EndoPAT Measurement

EndoPAT was determined using the Endo-PAT2000 device (Itamar, Caesarea, Israel) which measures the EndoPAT signal at the fingertip by recording arterial pulsatile volume changes [14,20,21]. These volume changes are the basis for the RHI; the automatically calculated ratio between post- and pre-occlusion EndoPAT signals corrected for the baseline vascular tone of the occluded arm. In addition, arterial stiffness (augmentation index, AIx) was automatically calculated from the EndoPAT signals in the baseline region of interest. AIx is estimated through the contribution of the reflected pressure wave to the systolic pressure signal impulse. AIx is defined as the difference between the peak caused by ventricular contraction and the second peak (augmentation pressure) of the arterial waveform. AIx is expressed as a percentage of the pulse pressure [22]. Each EndoPAT reading consisted of baseline readings for 5 min followed by 5 min of radial artery occlusion (cuff inflated at 250 mmHg systolic blood pressure) and reactive hyperaemia readings taken for 5 min after the release from occlusion. All EndoPAT measurements were performed on the left arm.

### 2.5. Office Blood Pressure Measurement

Office blood pressure was assessed according the guidelines of the International Society of Hypertension [23] at screening and prior to each set of vascular function measurements with an automatic arm cuff method using Omron blood pressure (BP) monitors (Omron Healthcare Europe BV, Hoofddorp, The Netherlands). To prevent a possible effect of the blood pressure measurement on the EndoPAT measurement, blood pressure was measured on the right arm. Heart rate was recorded by the blood pressure monitor as well. Each measurement comprised of three successive measurements within 10 min. The mean of the last two measurements was calculated and used in this study. The subjects were blinded for all blood pressure results during the study.

### 2.6. Urine Collection and Metabolic Profiling

Urine was collected during 24 h at all visit days in the period from entering the test facility to the next day for a profiling of fermentative metabolites by using the analytical techniques gas chromatography-mass spectrometry (GC-MS), liquid chromatography-multiple reaction monitoring-mass spectrometry (LC-MRM-MS) and NMR. The storage flask contained phosphoric acid (phosphoric acid gel—6 mL 50% MPA (w/w)) to optimally preserve the metabolites and to prevent bacterial contamination.

The GC-MS method focusing on the untargeted analysis of phenolic acids present in urine—including peak identification and quantification—has been described previously by Grün *et al.* [24]. The urine extracts were measured by GC-MS on an Agilent 5975C Mass Selective Detector (Agilent, Amstelveen, The Netherlands) equipped with an Agilent 7683B autosampler (Agilent, Amstelveen, The Netherlands) and an Agilent 7890A gas chromatograph (Agilent, Amstelveen, The Netherlands). The LC-MRM-MS method for the targeted detection and quantification of several catechins, and some other intact flavonoids as well as two phenylvalerolactones in urine was similar to the method described previously by Van Velzen *et al.* [25]. The isolation procedure includes an enzymatic deconjugation using beta-d-glucuronidase type 5 containing a high level of sulphatase, which was incubated for 2 h at 50 °C to remove glucuronic acid and sulphate conjugates. Deconjugated polyphenols were isolated by liquid-liquid extraction using ethyl acetate. For quantification, a triple quadrupole mass spectrometer in MRM mode equipped with an HPLC was used. Individual calibration curves ranging from 1 to 500 ng/mL were constructed for each polyphenol. Limits of detection defined as a signal-to-noise ratio better than 3 were 1 ng/mL for most polyphenols. The limits of quantification were experimentally estimated at 10 ng/mL for all polyphenols. The reproducibility was below 15% for all metabolites determined by LC-MRM-MS.

One-dimensional ^1^H NMR spectra of urine samples were acquired on a Bruker Avance 600 NMR spectrometer using a one-dimensional nuclear Overhauser enhancement spectroscopy (NOESY) pulse sequence with pre-saturation additional gradient dephasing of the intense water resonance during the relaxation delay of 3 s and the mixing time of 0.15 s (noesygppr). A 5-mm Triple Axis Inverse (TXI) resonance probe was used which was tuned to detect ^1^H resonances at 600.13 MHz. The internal probe temperature was set to 300 K. The spectral data were obtained in 32 K data points, a relaxation delay of 3 s and 128 transients. An exponential window function was applied to the free induction decay with a line-broadening factor of 0.5 Hz prior to the Fourier transformation. The FT NMR spectra were manually phase and baseline corrected and referenced to the sodium-3-(trimethylsilyl)-2,2,3,3-tetra-deuterioproprionate (TSP) resonance at 0.0 ppm using Topspin 1.3 software (Bruker Biospin GmbH, Rheinstetten, Germany).

Hippuric acid levels in urine were quantified from the NMR peak integral of the aromatic hippuric acid resonance at 7.83 ppm, relative to that of the internal standard TSP and expressed in g/24 h. In addition also the ratio of the hippuric acid to creatinine (HA/Crn) was calculated, using the creatinine-NCH3 signal at 3.10 ppm.

### 2.7. Statistical Analysis

All inferential statistical analysis of the microcirculation measurement and of all individually identified and quantified metabolites from urine measurements was performed using the SAS software package (SAS Institute, Cary North Carolina, version 9.1, Cary, NC, USA). All the related statistical tests were conducted against a two-sided alternative hypothesis, employing an overall significance level (α) of 0.05.

To focus visualizations on the main comparison of interest, data is shown either as change from baseline (value at *t* = 0 h) for time courses or change from placebo for dose response curves. The average of treatment in time is based on a covariance model (ANCOVA—analysis of covariance). The ANCOVA model included treatment as fixed effect and subject as a random effect in the model. Additional adjusting variables were examined and left in the model if significant at the 0.10 significance level. These additional adjusting variables included baseline values, visit, weight, age and sex. The *p*-values comparing individual doses against placebo were adjusted for multiple comparisons applying the Dunnet-Hsu method. Data are presented as LS means and 95% CIs.

### 2.8. Multivariate Analysis

The untargeted GC-MS and NMR metabolic profiles were analysed using multivariate data analysis in order to detect metabolic differences between theaflavin or catechin treatment groups and placebo. Binned profiles were imported into the multivariate statistical software package SIMCA-P version 12.0 (Umetrics, Umeå, Sweden). The metabolic profiles were subjected to a Principal Component Analysis (PCA) for examining the main sources of variation in the data [26]. Orthogonal Partial Least Squares regression to Latent Structures (O-PLS) was then performed, an extension to regular PLS-Discriminant Analysis (PLS-DA) to focus specifically on the separation between samples from different treatments. O-PLS-DA models for the pair-wise comparison of treatment and placebo samples were calculated, using one predictive component and one orthogonal component. The GC-MS and NMR O-PLS-DA models were validated using a 7-fold cross-validation and permutation testing [24,27].

## 3. Results

Twenty-four subjects started the study. One subject dropped out on measurement day 4, due to medical reasons (influenza), but recovered fully and data were collected at all other visits and included in the statistical analysis. This person was not replaced. One subject dropped out before the third visit day due to medical reasons and was not replaced. Data collected on the first 2 measurement days were included in the statistical analyses. Twenty-three subjects completed the study. The baseline characteristics of all 24 subjects are listed in Table 1.

**Table 1:** Baseline characteristics.

Characteristics	Value
Female	9 (37.5%)
Male	15 (62.5%)
Age, years	63 ± 6
Height, m	1.72 ± 0.09
Mass, kg	77.9 ± 11.0
BMI, kg/m^2^	26.4 ± 2.1
Seated office measurement	
SBP, mmHg	134.5 ± 14.3
DBP, mmHg	74.6 ± 9.4

BMI, body mass index; DBP, diastolic blood pressure; SBP, systolic blood pressure. Values are means ± SD or *n* (percentage).

**Figure 1:**
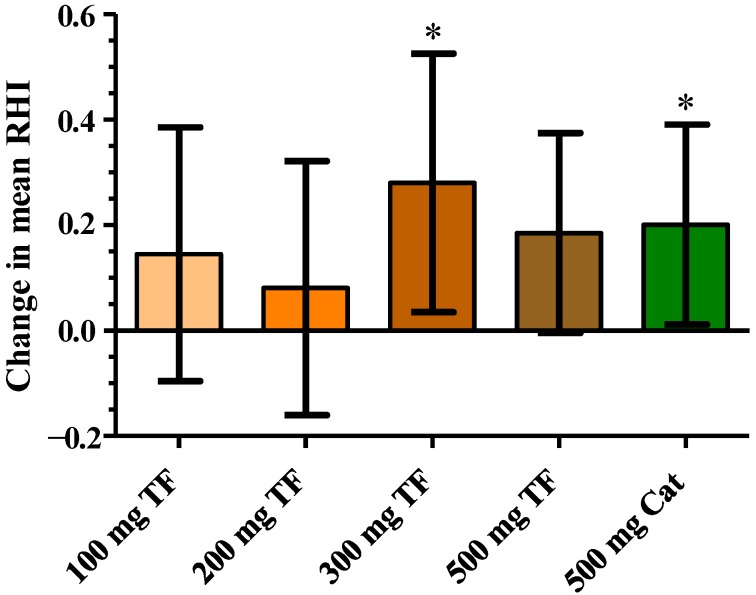
Effect of 100, 200, 300 and 500 mg theaflavins (TF) and 500 mg catechin (Cat) on reactive hyperemia index (RHI); data are presented as change to placebo of the mean of time points *t* = 2, 4 and 6 h after test product intake with *t* = 0 as covariate. Values are expressed as LSmeans and 95% CIs. * p < 0.05 compared to placebo.

The microcirculatory dilation measured as RHI tended to increase after the treatment with all theaflavins doses tested (Figure 1). The average effect calculated as the mean of the time points *t* = 2, 4 and 6 h after test product ingestion and corrected for baseline values revealed an increase in RHI with 500 mg theaflavins by 0.19 which was not statistically significant (*p* = 0.056). In addition, a modest but significant improvement of the RHI was observed after the consumption of 300 mg theaflavins (RHI = 0.28; *p* = 0.02). No effects were observed at lower doses. A dose-response effect of the varying doses was not observed and time kinetics did not reveal a clear maximal response time for theaflavins, which differed between theaflavins doses (data not shown). The intervention with 500 mg green tea catechins resulted in a small but significant increase in RHI (RHI = 0.20; *p* = 0.04). The improvement of RHI caused by the green tea catechins was in the same order of magnitude as that of 500 mg theaflavins. The effects on the RHI as measured by EndoPAT are illustrated in Figure 1 and Table 2.

**Table 2:** Vascular function in healthy subjects after the consumption of a single dose of 100, 200, 300 and 500 mg theaflavins and 500 mg catechins.

Outcome	Treatment				
	100 mg Theaflavin	200 mg Theaflavin	300 mg Theaflavin	500 mg Theaflavin	500 mg Catechins
Change in mean RHI	0.145	0.081	0.280 *	0.185	0.201 *
	(–0.095, 0.386)	(–0.160, 0.322)	(0.035, 0.525)	(–0.004, 0.375)	(0.012, 0.391)
Change in mean AIx	1.745	2.687	0.458	1.475	0.370
	(–2.855, 6.345)	(–1.920, 7.294)	(–4.176, 5.091)	(–3.123, 6.072)	(–3.755, 4.495)

RHI, reactive hyperemia index; AIx, augmentation index. Data are presented as change to placebo of the mean of time points *t* = 2, 4 and 6 h after test product intake with *t* = 0 as covariate. Values are expressed as LSmeans and 95% CIs. * *p* < 0.05 compared to placebo.

**Figure 2:**
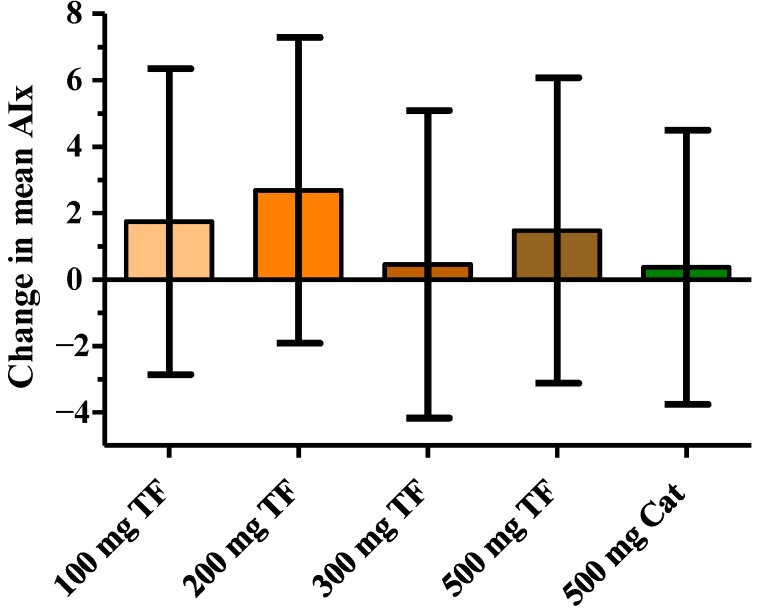
Effect of 100, 200, 300 and 500 mg theaflavins (TF) and 500 mg catechin (Cat) on augmentation index (AIx); data are presented as change to placebo of the mean of time points *t* = 2, 4 and 6 h after test product intake with *t* = 0 as covariate. Values are expressed as LSmeans and 95% CIs.

The AIx was calculated on the basis of EndoPAT data and is considered to be a marker for the arterial stiffness. Neither theaflavins nor the green tea catechins led to a change of the AIx (Figure 2, Table 2).

**Figure 3:**
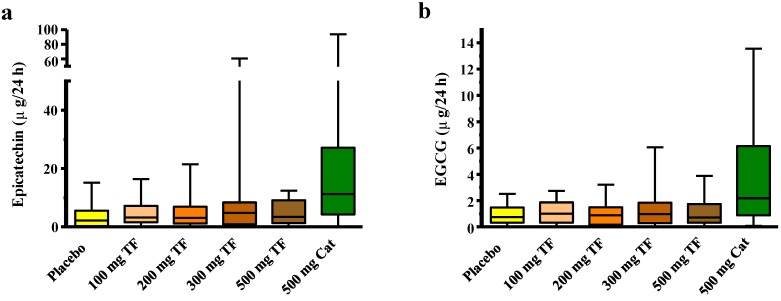
Boxplots (interquartile range, whiskers are min to max) of theaflavins (TF) or catechins (Cat) treatment related changes in (**a**) epicatechin and (**b**) epigallocatechingallate (EGCG) 24-h urinary excretion in all individuals as measured by targeted LC-MRM-MS.

The analysis of the 24-h urine by LC-MS demonstrated that the concentration of the catechins epicatechin, epigallocatechingallate (Figure 3; Table 3) and the catechin metabolites 3,4-dihydroxyphenyl-γ-valerolactone, 3′-*O*-methyl 3′,4′-dihydroxyphenyl-γ-valerolactone, gallic acid (all from targeted LC-MS) was significantly higher after consumption of 500 mg catechins compared to theaflavins or placebo ingestion. This was confirmed by untargeted multivariate data analysis of NMR and GC-MS metabolic profiles. In addition, pyrogallol was found to discriminate the green tea catechin intervention from placebo and black tea theaflavins. However, theaflavins were not detectable in the 24 h urine and the levels of metabolites of 40 other evaluated metabolites by the different platforms including the hippuric acid levels NMR were not significantly increased among others due to large individual variation. RHI effect size of theaflavins and the concentrations of the metabolites were not related (data not shown).

Absolute changes are expressed as LSMeans and 95% confidence intervals after correction for baseline.

The compliance of the test product was 100% as the test products were consumed at the study site. The compliance to the background diet was high, with non-compliance ranging from e.g., one cup of English tea to not having lunch. The analysis results of the 24 h urine indicate as well that subjects refrained from consuming polyphenol-rich products during the 24 h period. 

**Table 3:** Treatment related changes in urinary epicatechin and epigallocatechingallate (EGCG) levels in all individuals as measured by targeted LC-MRM-MS.

Catechin	Treatment					
	Placebo	100 mg Theaflavin	200 mg Theaflavin	300 mg Theaflavin	500 mg Theaflavin	500 mg Catechins
Epicatechin (μg/24 h)	3.53	5.18	4.83	8.43	4.95	22.47
	(1.78, 1.78)	(3.10, 7.25)	(2.65, 7.00)	(2.55, 14.31)	(3.13, 6.77)	(10.25, 34.69)
EGCG (μg/24 h)	0.92	1.097	0.96	1.39	1.02	3.75
	(0.60, 1.25)	(0.72, 1.47)	(0.56, 1.37)	(0.73, 2.05)	(0.63, 1.41)	(2.13, 5.37)

## 4. Discussion

The present study was the first investigating the acute effects of black tea theaflavins on (micro-) vascular function, and we demonstrated that at a single dose of theaflavins improves microvascular reactive hyperemia. The observed effect size did not significantly differ from green tea-derived catechins, which is in line with the effects of black and green tea on endothelial function as described previously [15].

Clinical studies conducted with pure flavonoids on vascular health parameters are scarce. Schroeter *et al.* showed that the pure flavonoid (−)-epicatechin exerts effects on vascular function determined by FMD and EndoPAT measurements [16,19]. Intake of epicatechin acutely augmented plasma nitric oxide status and decreased endothelin-1 concentration [28]. Acute but not chronic effects on FMD of epigallocatechingallate (EGCG) have been reported [29]. Other investigators have particularly focused on the effects of flavonoid-rich or enriched food products, such as tea [5,14], cocoa [18], apple [30] or wine [31]. The potential blood pressure lowering effects of cocoa have been summarized in a systematic review [32] and a recent meta-analysis confirmed a similar reduction in blood pressure by regular tea intake [33]. The overall picture seems to be that flavonoids are important mediators in the vascular benefits, but it remains ambiguous whether certain flavonoids or classes of flavonoids contribute more than others. We have identified possible tea flavonoids contributing to improving vascular function, but the increase of reactive hyperemia exerted by theaflavins and catechins in the current study was modest. A dose-dependency as observed for black tea on large vessel function [34] has not been found for black tea theaflavins in the current study. There was also no common pattern over time in the effects of the varying theaflavin doses. Moreover, the pattern of the effect of catechin on RHI over time differed from that described in the literature. Schroeter *et al.* reported the maximum vascular response measured by EndoPAT 2 h after the intake of (−)-epicatechin [19] while the maximum effect of green tea catechins in the current study was observed 4 hours after the intake of the catechin capsules.

Another outcome of the EndoPAT measurement was the augmentation index (AIx). While theaflavins and catechins caused at least a modest improvement of the RHI, the AIx was not affected by any treatment which is probably due to the acute setting of the study. Chronic intervention periods may be required to influence arterial stiffness.

The test products have been ingested at the study site. The results of the 24 h analyses by GC-MS, LC-MS and NMR confirmed a good compliance of the subjects to the study protocol including the background diet as the level of catechin metabolites was only increased after intake of 500 mg green tea catechins.

The urinary analysis confirmed the appearance of well-known catechin metabolites (pyrogallol, valerolactones, gallic acid), but surprisingly after theaflavins intake no metabolites could be identified. Due to the relatively large molecular weight of theaflavins, bioavailability of these molecules was as expected very low, and basically not detectable in urine.

Due to the limited knowledge on the metabolism of theaflavins the current explorative study has some limitations. Microbial metabolites of theaflavins may have been formed, appearing in the blood after the vascular measurements. Effects of these metabolites as well as effects of theaflavins after chronic consumption were not addressed in the current acute study design. The EndoPAT technique provides data with a very high reproducibility, but the exact location of the vascular bed evaluated by this technique still remains unclear, also because it represents a response of ischemic tissue that integrates endothelium-dependent and -independent mechanisms [11]. Effects of theaflavins should therefore be confirmed with other tools that assess vascular function.

## 5. Conclusions

The study indicates that catechines and theaflavins may contribute to the vascular effects of green and black tea, respectively. The moderate effects suggest that other components in tea may also play a role in affecting vascular function. Therefore, more research is required to explain why green tea and black tea show similar sized effects on vascular function despite the differences in composition.
